# Supplementary material for: Validation and application of the Chinese version of the Perceived Stress Questionnaire (C-PSQ) in nursing students
Source: PeerJ. 2018 Mar 8;6:e4503. doi: 10.7717/peerj.4503 (PMC5845571; doi:10.7717/peerj.4503)
Supplement: Supplemental Information 2 [file peerj-06-4503-s002.pdf]

General PS Questionnaire

For each sentence, mark the number that describes how often it applies to you *in general, during the last two years*. Work quickly, without bothering to check your answers, and be careful to describe your life *in the long run*.

		Almost never 1	Some-times 2	Often 3	Usually 4
01	You feel rested				
02	You feel that too many demands are being made on you				
03	You are irritable or grouchy				
04	You have too many things to do				
05	You feel lonely or isolated				
06	You find yourself in situations of conflict				
07	You feel you're doing things you really like				
08	You feel tired				
09	You fear you may not manage to attain your goals				
10	You feel calm				
11	You have too many decisions to make				
12	You feel frustrated				
13	You are full of energy				
14	You feel tense				
15	Your problems seem to be piling up				
16	You feel you're in a hurry				
17	You feel safe and protected				
18	You have many worries				
19	You are under pressure from other people				
20	You feel discouraged				
21	You enjoy yourself				
22	You are afraid for the future				
23	You feel you're doing things because you have to, not because you want to				
24	You feel criticized or judged				
25	You are lighthearted				
26	You feel mentally exhausted				
27	You have trouble relaxing				
28	You feel loaded down with responsibility				
29	You have enough time for yourself				
30	You feel under pressure from deadlines				
