# Supplementary material for: Validation and application of the Chinese version of the Perceived Stress Questionnaire (C-PSQ) in nursing students
Source: PeerJ. 2018 Mar 8;6:e4503. doi: 10.7717/peerj.4503 (PMC5845571; doi:10.7717/peerj.4503)
Supplement: Supplemental Information 4 [file peerj-06-4503-s004.pdf]

简体中文版《压力感知问卷》(C-PSQ)

请根据您***过去两年间***的总体感受，在每个句子后标出最符合的选项（框内画“ √ ”）。
请依次快速作答，不必反复核实，并注意从***长期***的角度来描述。

	几乎从未	有时	时常	通常
01. 你感到精神焕发。	①	②	③	④
02. 你感觉你被要求过多。	①	②	③	④
03. 你急躁易怒或好发牢骚。	①	②	③	④
04. 你要做的事情太多了。	①	②	③	④
05. 你感到孤独或被孤立。	①	②	③	④
06. 你发现自己处于矛盾冲突的境地。	①	②	③	④
07. 你认为你在做自己真正喜欢的事情。	①	②	③	④
08. 你感到疲惫。	①	②	③	④
09. 你害怕自己可能不去设法实现你的目标。	①	②	③	④
10. 你感到镇定平静。	①	②	③	④
11. 你有很多决定需要做。	①	②	③	④
12. 你感到挫败。	①	②	③	④
13. 你感到充满精力。	①	②	③	④
14. 你感到紧张。	①	②	③	④
15. 你的问题似乎越堆越多。	①	②	③	④
16. 你觉得自己很匆忙。	①	②	③	④
17. 你感到安全并受到保护。	①	②	③	④
18. 你有许多担忧。	①	②	③	④
19. 你承受着来自别人的压力。	①	②	③	④
20. 你感到灰心。	①	②	③	④
21. 你有享受到乐趣。	①	②	③	④
22. 你对未来感到恐惧。	①	②	③	④
23. 你认为你正在做的事情，是因为你必须做而不是你想要做。	①	②	③	④
24. 你感到被批评或评判。	①	②	③	④
25. 你过得无忧无虑。	①	②	③	④
26. 你觉得精神上很疲惫。	①	②	③	④
27. 你很难放松下来。	①	②	③	④
28. 你觉得责任使你负担过重。	①	②	③	④
29. 你有足够的时间给自己。	①	②	③	④
30. 截止日期让你承受着压力。	①	②	③	④

Author: Susan Levenstein, M.D.; Translator: Runtang Meng, Ph.D. Candidate
C-PSQ-G, Last updated December 2016
